# Repellent Activity of Botanical Oils against Asian Citrus Psyllid, *Diaphorina citri* (Hemiptera: Liviidae)

**DOI:** 10.3390/insects7030035

**Published:** 2016-07-14

**Authors:** Emily H. Kuhns, Xavier Martini, Angel Hoyte, Lukasz L. Stelinski

**Affiliations:** 1 Citrus Research and Education Center, Entomological and Nematology Department, University of Florida, 700 Experimentation road, Lake Alfred, FL 33850, USA; emilyhkuhns@gmail.com (E.H.K.); xmartini@ufl.edu (X.M.); ahoyte@ufl.edu (A.H.); 2 North Florida Research and Education Center, Entomological and Nematology Department, University of Florida, 155 Experiment Road, Quincy, FL 32351, USA

**Keywords:** citrus greening disease, essential oil, repellent, push-pull, integrated pest management

## Abstract

The Asian citrus psyllid, *Diaphorina citri* Kuwayama, is the insect vector of the pathogen causing huanglongbing. We selected three botanical oils to evaluate behavioral activity against *D. citri*. In laboratory olfactometer assays, fir oil was repellent to *D. citri* females, while litsea and citronella oils elicited no response from *D. citri* females. In choice settling experiments, *D. citri* settled almost completely on control plants rather than on plants treated with fir oil at a 9.5 mg/day release rate. Therefore, we conducted field trials to determine if fir oil reduced *D. citri* densities in citrus groves. We found no repellency of *D. citri* from sweet orange resets that were treated with fir oil dispensers releasing 10.4 g/day/tree as compared with control plots. However, we found a two-week decrease in populations of *D. citri* as compared with controls when the deployment rate of these dispensers was doubled. Our results suggest that treatment of citrus with fir oil may have limited activity as a stand-alone management tool for *D. citri* and would require integration with other management practices.

## 1. Introduction

The Asian citrus psyllid, *Diaphorina citri* Kuwayama, is the vector of the causal agent of the pathogens causing huanglongbing (HLB). HLB is the most damaging disease of citrus worldwide. HLB, also known as citrus greening disease, causes yellowing of shoots, stem dieback, sour fruit, crop losses and eventually tree death [1,2]. The causal agent of this disease in the USA is presumed to be the bacterium, *Candidatus Liberibacter asiaticus* (*C*Las) [3], and is transmitted from plant to plant through the movement and feeding of adult *D. citri* [4]. Found for the first time in 1998 [5], *D. citri* has now spread throughout Florida and has been impossible to eradicate. The impacts of HLB on the Florida economy are dramatic. Approximately 80% of citrus is now infected with the pathogen [6] and the 2015–2016 citrus yield in Florida decreased by approximately 30% compared to the previous year, and is the worst season for this citrus industry in 50 years. 

Current methods to prevent the spread of HLB rely heavily on management of the psyllid vector. Typically, insecticides are used to manage populations of *D. citri* [7,8], which has led to insecticide resistance [9] and negative impacts on populations of natural enemies [10]. While resistance to insecticides can be managed by rotation of modes of action through time and space [11], these chemicals often have undesired non-target effects and their use is limited by application restrictions to food crops [12]. Additionally, there are few options available to organic citrus growers to manage *D. citri*. Therefore, alternative pest control tools will be valuable for supplementing existing insecticide-based management programs. Pest control using botanical oils to affect insect behavior can be complementary to insecticide use, because the oils should not interfere with the insecticide mode of action. Botanical oils can disrupt host insect interactions by repelling insects from a host, deterring feeding, or by causing contact toxicity [13]. In addition, botanical oils are considered safe for many food crops and are often acceptable in organic farming, making them a versatile pest management option when traditional insecticides are not acceptable [13].

Botanical oils have been evaluated as potential repellents for several insect orders including: Lepidoptera [14], Diptera [15], and Coleoptera [16]. For *D. citri*, a number of botanical oils have been evaluated in laboratory assays, including those from tea tree, thyme, lavender, rose, and coriander [17]. While some of those oils showed promising results in laboratory bioassays, the costs of the active ingredients prohibited development of formulations for use in the field [17]. 

In this investigation, we evaluate previously untested botanical oils for behavioral activity (repellency) against *D. citri* in the laboratory. Based on these results, we then only tested the most active oil under field conditions as a possible management tool for this pest. Fir, litsea, and citronella oils were chosen because they are widely available, considered inexpensive, and from non-Rutaceae plants (non-hosts). Our objectives were to: (1) test these oils for repellency to *D. citri* in laboratory olfactometer bioassays; (2) determine if behaviorally active oil(s) deter settling of *D. citri* onto seedling citrus in caged settling experiments; and (3) test oil(s) that were effective in laboratory experiments under field growing conditions. Given that fir oil was the only treatment to cause repellency of *D. citri* in olfactometer bioassays (see Results), this was the only oil investigated further in settling and field experiments. 

## 2. Materials and Methods 

### 2.1. Chemicals 

Ethylene glycol (≥99% purity, item # 102466), Canadian fir needle (item # W523518) and Chinese citronella (item # W230804) oils were purchased from Sigma Aldrich (St. Louis, MO, USA). Chinese *Litsea cubeba* oil was purchased from New Directions Aromatics (Brampton, ON, Canada). High release fir oil devices for use in field trials were purchased from Alpha Scents (West Linn, OR, USA).

### 2.2. Odor Release Dispensers 

Botanical oils were dispensed by diffusion through polyethylene containers. Fir oil was released using 0.6 mL polyethylene BEEM vials (SPI Supplies, BEEM #1001, Size 00, Westchester, PA, USA). For the ‘lab-low’ dose, 100 µL of undiluted fir oil was pipetted into each vial and the lid was closed to obtain an average release rate of 5 mg/day (see Results). For the ‘lab-high’ dosage, the vials were prepared as for the low dosage, but a 1 mm hole was punctured in the lid to obtain an average release rate of 9.5 mg/day (see results). 

For the high release rate of fir oil used in field trials (‘field-high’), dispensers were made by Alpha Scents. These field dispensers consisted of large polyethylene ziplock style bags that were heat-sealed and allowed for slow diffusion through the polyethylene matrix. The average release rate for these field dispensers was 2.6 g/day (see Results). Control release dispensers used in the field were empty and consisted of polyethylene. 

The release rates for each type of dispenser were measured gravimetrically with a balance (Veritas, Model S622, sensitivity: 0.01 g) to determine evaporation of the active ingredient over time. Only the release rate of fir oil from dispensers was measured, because this was the only treatment to affect behavior of *D. citri* in the initial laboratory assays (see Results). The ‘lab-low’ and ‘lab-high’ fir oil dispensers were hung in a laboratory fume hood at 26 °C and weighed daily for 4 days after deployment. There were five replications per dispenser treatment per interval measured. 

The ‘field-high’ fir oil dispensers were deployed outdoors in Lake Alfred, FL (28.1′N, 81.7′W) under similar conditions to the field trials in 2013. These were weighed for the first 5 consecutive days and then weekly thereafter for 30 days. Five replicates of these dispensers were weighed during each time period. For each of the release disperser types, the average release rate was calculated and the data were fitted with exponential decay curves (see Results).

### 2.3. Psyllid Rearing 

For the olfactometer assays, *D. citri* were obtained from a laboratory culture maintained at the University of Florida, Citrus Research and Education Center (Lake Alfred, FL, USA). The culture was established in 2000 from field populations in Polk Co., FL, USA (28.0′N, 81.9′W), prior to the discovery of HLB in Florida. The culture was maintained without exposure to insecticides on Valencia sour orange (*Citrus aurantium* L.) in an air-conditioned greenhouse at 27–28 °C, 60%–65% r.h., and L14:D10 photoperiod. Bi-monthly testing of randomly sampled nymphs, adults, and plants by quantitative polymerase chain reacton (qPCR) assays were conducted to confirm that psyllids and plants in this culture were uninfected by *C*Las.

The *D. citri* population used for settling bioassays was established from wild *D. citri* adults collected in July 2012 from Winter Garden, FL (28°28′9.70″N 81°39′23.46″W) and Lake Alfred and subsequently housed in a climate controlled room with a temperature of 25–28 °C, a photoperiod of L14:D10, and a relative humidity of 40% ± 5%. This population was maintained for the duration of the laboratory experiments (approximately three months) and allowed to reproduce freely. The *D. citri* culture was sustained in a 0.4 × 0.4 × 0.75 m screen-rearing cage (BioQuip, Rancho Dominguez, CA, USA) containing 10 uninfected two-year-old sweet orange (*Citrus sinensis*) seedlings. The plants were fertilized with Young Tree Citrus Special granular fertilizer, N-P-K ratio of 6-4-6 (Growers Fertilizer Corporation, Lake Alfred, FL, USA) every 21 days according to manufacturer’s suggestions.

### 2.4. Olfactometer Assays 

Olfactometer assays were performed between 0900 and 1300 h, using a custom made, two-arms, T-olfactometer from Analytical Research Systems (Gainesville, FL, USA), as described in Mann et al. [18]. Experiments were conducted in a climate-controlled laboratory (25 °C and 40% RH, L14:D10 photoperiod) with overhead fluorescent lighting set to a 16:8 light cycle. To ensure chemical free ambient air supply, both arms of the olfactometer received charcoal-purified and humidified air from a custom made air delivery system (ARS, Gainesville, FL, USA). A constant airflow of 0.1 L·min^−1^ was maintained through both arms of the olfactometer. The olfactometer was positioned vertically under a fluorescent 23 W light source (FLE23HT3/3/SW, GE Lighting, Cleveland, OH, USA) mounted within a 1.0 × 0.6 × 0.6 m fiberboard box for uniform light diffusion. Psyllids were acclimated to the environmental conditions of the laboratory for at least 30 min prior to initiation of experiments, and there was an approximate 2-h interval between the time when psyllids were removed from rearing and when they were assayed. Given their sensitivity to starvation [19], *D. citri* were not purposely starved prior to assays. *D. citri* of varying ages were sorted by gender prior to the assays and only females were used for the experiment given their greater response to olfactory cues than males [20]. A single *D. citri* female was inserted in the olfactometer per replicate, and was allowed up to 5 min to select an odor source. A positive response was scored when an adult *D. citri* traveled past the divider and into one of the two arms presenting an odor. Psyllids were considered non responders when they did not pass the divider after 5 min. Non responders were excluded from further statistical analysis. After each treatment, the glassware was cleaned with Sparkleen 1 detergent (Thermo Fisher Scientific, Waltham, MA, USA) followed by an acetone wash before placing in a drying oven at 60 °C for a minimum of 1 h.

The botanical oils were diluted to 1, 5, 15 or 30 mg in 100 µL of ethylene glycol. Ethylene glycol alone was used as the control. One hundred microliters of the oil mixtures were applied to a one-inch cotton wick and wrapped in a Kimwipe laboratory tissue (Kimberly-Clark, Roswell, GA, USA) and placed in the upstream odor chambers apposing 100 µL of ethylene glycol only (Table 1). Citrus odors were obtained by clipping 4 g of crushed Valencia sour orange leaf flush from citrus plants maintained in the laboratory greenhouse and similarly wrapped in laboratory tissue for placement into the treatment chambers (Table 2). Responses of *D. citri* to clean air vs. ethylene glycol, as well as citrus leaf volatiles with and without ethylene glycol were measured as additional controls to ensure no bias in the olfactometer prior to evaluation of treatments.

When testing a botanical oil treatment with intact (non-damaged) citrus plants, individual Valencia sour orange plants (20–30 cm height) were enclosed within custom made two-port glass domes (40 cm height, 15 cm ID). The upper and lower portions of each plant were separated by a PTFE guillotine (2.5 cm width), so that the same amount of tree canopy was exposed to the airflow entering each arm of the olfactometer as described in [21]. The guillotine can be opened to insert the plant into the glass dome chamber without damage. The plants were positioned under a 150 W high-pressure sodium grow light (Hydrofarm, Petaluma, CA, USA). 

The glass dome was equipped with an inlet for entry of airflow from the air delivery system and an outlet for pushing air to the connected chamber containing either ethylene glycol or the repellent odor-treated cotton wick (treatment). For initial screening, we tested each concentration of fir, litsea, and citronella oils against ethylene glycol. In addition, we tested the repellency of fir oil with crushed citrus against crushed citrus alone, as well as fir oil with an intact citrus plant against an intact citrus plant alone. We assayed between 57 and 119 individual *D. citri* per treatment for each of the two experiments. Pools of approximately 30 individuals were tested per treatment combination per day. Only one treatment combination was tested daily and chosen at random. For some treatment combinations more than 30 *D. citri* were assayed when adults were abundant. 

### 2.5. Settling Bioassays

Seeds from local *C. sinensis* var. Valencia were planted in a greenhouse. These seedlings were kept free of pests and disease manually. They were fertilized with Growers Young Tree Citrus Special (Growers Fertilizer Corporation, Lake Alfred, FL, USA) with an N-P-K ratio of 6-4-6 every 21 days and a liquid, all-purpose fertilizer (Scott’s Miracle Gro, water-soluble fertilizer, Marysville, OH, USA) with an N-P-K ratio of 24-8-16 administered via Water-Powered Injector (Item #HS15-5, 14 GPM D14MZ3000-14, Dosatron International, Clearwater, FL, USA), at a 2% dose every 7 days. When the seedlings were approximately 1 year old and between 15 and 20 cm in height, they were used for settling assays in screen cages (0.75 × 0.4 × 0.4 m). Experiments were conducted in the same climate controlled laboratory (26 °C and 45% RH, L14:D10 photoperiod) with overhead fluorescent lighting set to a 16:8 light cycle. Two replicates were conducted at the same time, cages were spaced at 1 m. 

Each cage contained four seedlings. Within each screen cage, two seedlings were designated as controls and two seedlings were treated with fir oil. Control seedlings were placed adjacent to one another at one end of the cage and the fir oil treated seedlings were placed 50 cm away on the opposite end of the cage. The control seedlings were treated with an empty polyethylene vial, while the other two seedlings were treated with the ‘lab-high’ dose dispenser of fir oil. In total, one vial was applied to each plant. The ‘lab-high’ dispenser was assayed in these choice settling assays, because the ‘lab-low’ dosage had no effect on behavior of *D. citri* in previously conducted no-choice settling assays. 

Approximately one hundred field-collected *D. citri* in culture, adult *D. citri* of mixed age were released into the center of each cage and allowed to settle on seedlings. For practical reasons, psyllids used in this experiment were of mixed gender. Sex ratio in our *D. citri* rearing is 1:1. All *D. citri* that had settled on seedlings, or were found dead within the cages, were counted 24 and 72 h following release. The experiment was replicated six times.

### 2.6. GC-MS Sample Preparation

We examined the composition of fir oil. One microliter of fir oil was deposited into 40-mL glass vials that were sealed with tin foil. A triphase 50/30 µm DVB/Carboxen/PDMS StableFlex solid-phase microextraction (SPME) fiber for volatiles and semivolatiles with molecular weight between 40 and 275 (Supelco, Bellefonte, PA, USA) was inserted through the tin foil lid and exposed to the fir oil odor for 5 s. The SPME fiber was desorbed for 5 min at 240 °C under splitless conditions and the odor constituents were separated over 37 min on a Stabilwax (Restek, Bellefonte, PA, USA) capillary column (60 m × 0.25 mm inner diameter; 0.5 µm film thickness) using a temperature gradient from 40 to 240 °C at 7 °C/min, and hold at 240 °C for 8 min. Helium was used as a carrier gas at 2 mL/min. Identification of the compounds was performed using a Clarus 500 quadrupole mass spectrometer and turbo mass software (Perkin Elmer, Shelton, CT, USA). Linear retention times of authentic standards, when available, and mass spectra from the NIST database, were used to identify compounds.

### 2.7. Field Evaluation

The field experiments were conducted under commercial growing conditions in 2013 and 2014. We evaluated the ‘field-high’ dispensers, described above, from Alpha Scents as a repellent for *D. citri*. Both experiments were conducted in a sweet orange grove in Winter Garden, FL (40 km north from Lake Alfred where the release tests of the dispensers were conducted). The experiment was deployed in a 12 × 30 tree block of 4-year-old Valencia sweet orange reset trees. Five blocks of 12 (4 m tall × 3 m across) trees were treated with fir oil (*n* = 5) or negative controls (empty dispensers) (*n* = 5). Treatments were assigned randomly to each block. Four ‘field-high’ fir oil dispensers or blank controls were hung within the canopy of each treated tree in 2013. These blocks were arranged as two vertical rows of trees with two row buffers (16 m) separating blocks. No insecticides were applied to the entire grove during the experiment. The grove was surrounded by resets of the same age and variety on the north and east side, while fallow fields were to the south and west. *D. citri* were counted by tapping citrus branches using a 40 cm section of plastic pipe three times to dislodge insects onto a white laminated 21.6 × 28 cm paper surface for counting. Two tap counts were taken per tree from each plot. Tap counts of adult *D. citri* were conducted the day before the fir oil treatment, and weekly thereafter. 

The second field experiment was conducted in 2014 identically to that described above; however, the number of fir oil dispensers applied per tree was doubled in an effort to improve treatment efficacy. In this case, eight ‘field-high’ release devices were attached per tree and this was compared against a blank negative control (8 empty vials per tree). The location of the experiment, experimental design, and sampling protocol were identical to that described for 2013.

Weather data were obtained from the Florida Automated Weather Network (FAWN) stations located approximately 1 km from the experimental sites in Lake Alfred and Winter Garden, respectively.

### 2.8. Statistical Analysis

Chi square goodness of fit tests were used to determine significance of choice between botanical odors vs. the control in two-choice T-olfactometer assays (non-responder were excluded from this analysis). In the choice settling assays, a paired *t*-test was used to determine if there was a difference between the mean number of *D. citri* on blank control vs. ‘lab-high’ fir oil treated seedlings. To avoid pseudo-replication, both the 24 and 72-h time points were analyzed for statistical significance difference between treatments. For field trials, the averages of psyllids collected by tap sampling per tree within each plot were compared with *t*-tests. To avoid pseudo-replication, each time point was analyzed separately for statistical significance difference between treatments. Analysis was performed with SigmaPlot 13.0 (Systat Software, San Jose, CA, USA). *p*-Values of less than 0.05 were considered significant.

## 3. Results

### 3.1. Olfactometer Assays

*D. citri* females were significantly repelled by fir oil at both the 5 and 15 mg doses; however, their behavior was not affected by litsea oil or citronella oil vs. ethylene glycol (Table 1). In a subsequent experiment, 15 mg of fir oil was tested in the presence of crushed citrus against crushed citrus alone. In this case, the fir oil with crushed citrus did not repel *D. citri* females as compared with crushed citrus alone (Table 2). After modifying the assay to use intact, undamaged citrus seedlings in place of crushed citrus, we found that fir oil repelled *D. citri* at the 15 mg dose, but not at the 1 or 5 mg doses, as compared with undamaged citrus odor (Table 2). 

### 3.2. Settling Bioassays

**Figure 1:**
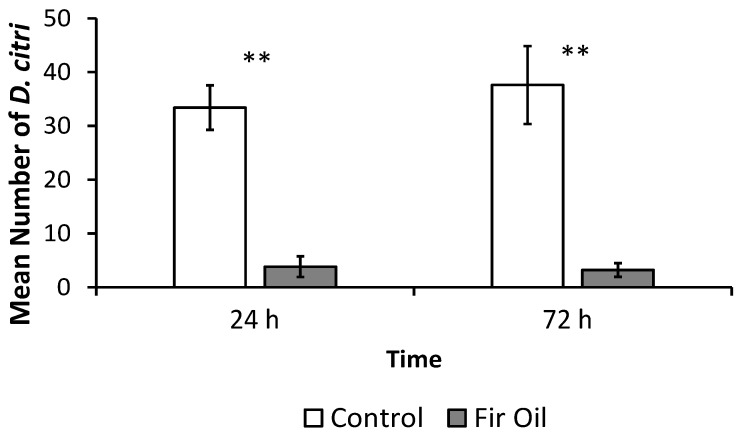
Mean (±SE) number of *Diaphorina citri* settling per plant on citrus seedlings treated with fir oil after 24 and 72 h as compared with blank control seedlings in a choice bioassays ** <0.01.

Fir oil was chosen for further testing since it repelled *D. citri* in the initial T-olfactometer screening tests. *D. citri* males and females were presented with a choice between control seedlings and ‘lab-high’ fir oil-treated seedlings. In the choice test, *D. citri* disproportionately settled on control seedlings as compared with lab-high fir oil treated seedlings at 24 (*t* = 6.34, df = 5, *p* = 0.001) and 72 h (Figure 1; *t* = 4.94, df = 5, *p* = 0.004). Although sex ratio was undetermined, percent repellency observed (10.10% on treated seedlings vs. 89.89% on control seedlings) was significantly different from a hypothetical scenario where only females would be repelled, but not males (expected 25 vs. 75%; *χ*^2^ = 24.64, df = 1, *p* < 0.001). This indicates that both male and female psyllids were repelled by fir oil during the experiment. GC-MS analysis indicated that the four major components of fir oil were α-pinene, 2-β-pinene, δ-3-Carene and camphene. These four components alone accounted for 78% of the fir oil (Table 3). 

### 3.3. Field Evaluation

**Figure 2:**
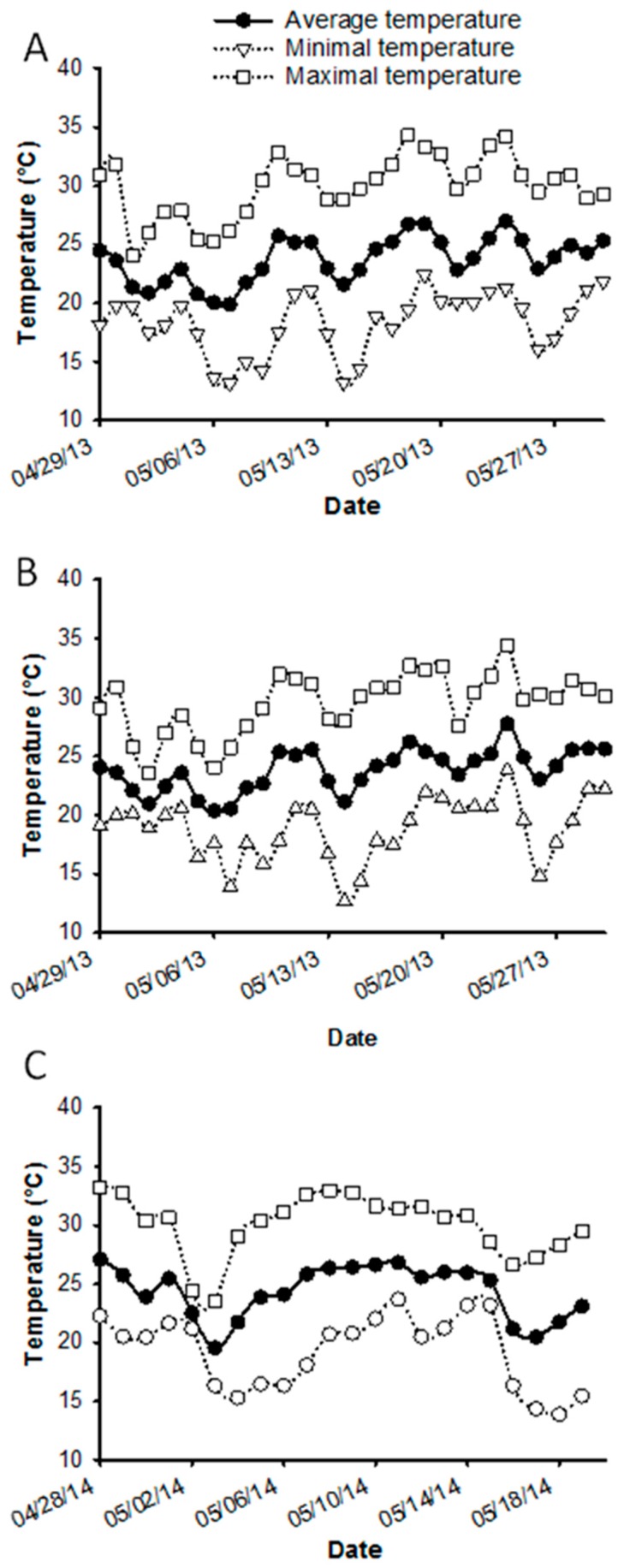
Average, minimal, and maximal temperatures recorded in Lake Alfred, FL during examination of dispenser release rates in 2013 (**A**); in Winter Garden, FL during 2013 (**B**) and 2014 (**C**) field trials of fir oil as a repellent for *D. citri*.

The average daily temperature in Lake Alfred in 2013 was 23.69 °C (min 13.14, max 34.31 °C) (Figure 2A) with an average relative humidity of 74%. In Winter Garden, the average daily temperature in 2013 was 24.00 °C (min 12.67, max 34.38 °C) (Figure 2B) with 75% average relative humidity. In 2014, the average temperature in Winter Garden was 24.33 °C (min 13.88, max 33.25 °C) (Figure 2C) with 74% relative humidity. Therefore, the conditions were fairly consistent during the two field trials and the measure of the release rate of the fir oil devices.

**Figure 3:**
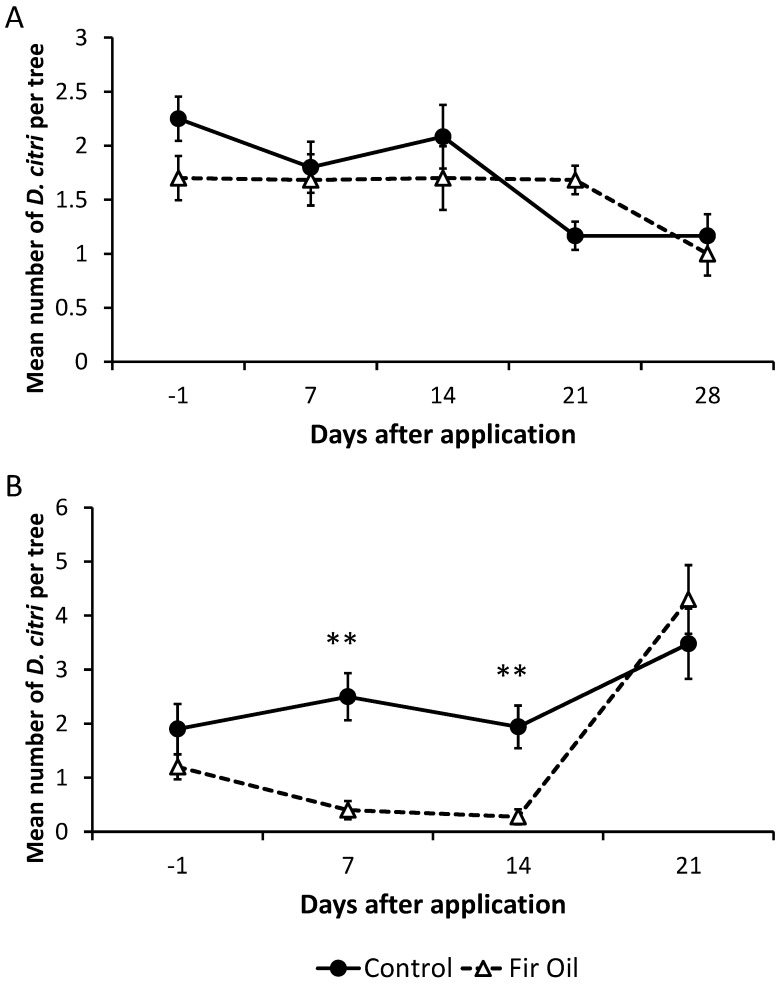
Mean (±SE) number of *Diaphorina citri* tap counted on fir oil-treated (4 dispensers/tree) vs. control plots in field trials for four weeks in 2013 (**A**) and on fir oil-treated (8 dispensers/tree) vs. control plots in field trials for three weeks in 2014 (**B**).

In 2013, there was a slight difference in the mean number of *D. citri* adults found between plots designated for treatment and control before treatments were applied (*t* = 2.339, df = 8, *p* = 0.048). However, there were no significant differences in the numbers of *D. citri* in plots treated with fir oil vs. the control at 7 (*t* = 0.472, df = 8, *p* = 0.650), 14 (*t* = 1.019, df = 8, *p* = 0.338), 21 (*t* = 1.833, df = 8, *p* = 0.104), and 28 days (*t* = 0.557, df = 8, *p* = 0.593) after dispensers with fir oil were deployed (Figure 3A). 

In 2014, the pre-treatment numbers of *D. citri* did not differ between plots designated for fir oil treatment vs. the control plots (*t* = 1.347, df = 8, *p* = 0.215). With the number of fir oil dispensers doubled (8 per tree), compared to 2013, the mean number of *D. citri* in treated plots was reduced significantly at 7 (*t* = 4.487, df = 8, *p* = 0.002) and 14 (*t* = 3.968, df = 8, *p* = 0.004) days after dispensers were deployed as compared with controls, but not at 21 days following deployment (*t* = 0.904, df = 8, *p* = 0.392) (Figure 3B). 

### 3.4. Release Rates

**Figure 4:**
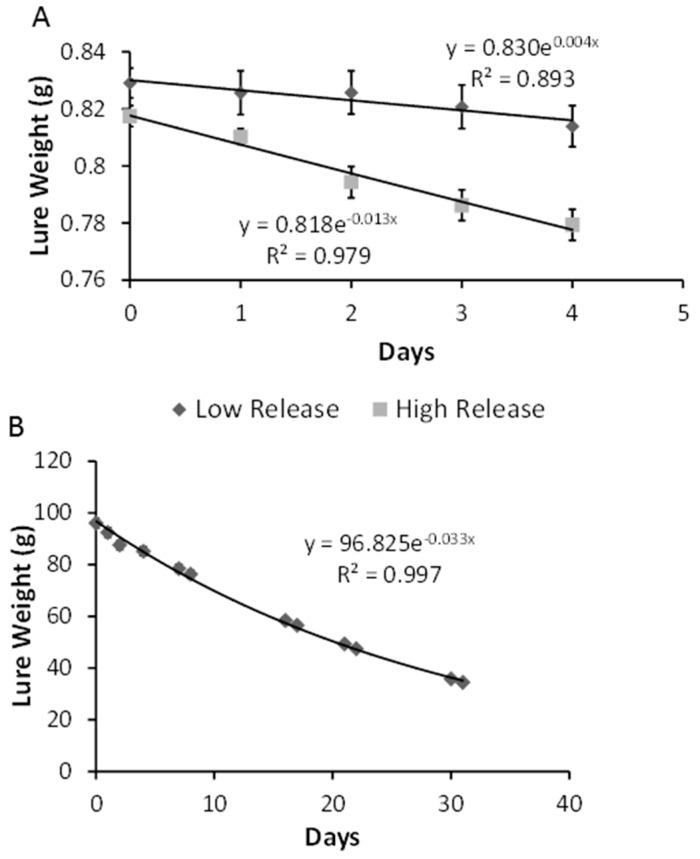
Exponential decay curves for botanical oil dispensers. Laboratory fir oil dispensers with (squares) and without (diamonds) a hole drilled into the lid (**A**); High release fir oil dispensers used in field trials (2013 only) (**B**).

The average gravimetric release rates for the ‘lab-low’ and ‘lab-high’ fir oil dispensers were estimated to be 5.1 ± 0.79 mg/day and 9.5 ± 1.33 mg/day, respectively (Figure 4A). The exponential decay constant was estimated to be −0.004 for the lab-low fir oil dispensers (*R*^2^ = 0.893) and −0.013 for the lab-high fir oil dispensers (*R*^2^ = 0.979). The ‘field-high’ fir oil released was on average 2.6 ± 0.25 g/day per dispenser for the duration of the experiment and the exponential decay constant was −0.033 (*R*^2^ = 0.997; Figure 4B).

## 4. Discussion

Insects rely on a variety of cues to identify acceptable host plants for mating and reproduction, including light intensity, color patterns, humidity, and nutritional value [22]. Volatile odor components of plants also play a pivotal role in host identification and acceptance in insects. For some insects, odor cues can be exploited to alter how insects perceive their landscape. Acceptable plant host odors, in combination with other environmental cues, can be attractive to insects while non-host odors can either repel insects or have no effect on behavior, allowing insects to navigate a complex landscape to find suitable hosts [23]. By using concentrated, non-host botanical odors, we attempted to compromise the ability of *D. citri* to find and infest a preferred host (citrus).

Fir needle oil repelled *D. citri* females in our initial olfactometer assays and repelled both sex in a two-choice settling bioassay. When we tested fir oil in the field, populations of *D. citri* were not affected at the deployment rate dosage tested in 2013. Four ‘field-high’ dispensers were placed per tree resulting in an estimated release of about 10.4 g of fir oil (4 dispensers releasing in average 2.6 g per day each) or just over 1000-fold the amount released in the laboratory. Based on the canopy volume and the abiotic conditions measured in the field vs. laboratory conditions, there should have been comparable release of fir oil from these dispensers. However, dissipation of volatiles was likely greater under field conditions than in a laboratory olfactometer. This may partially explain why populations of *D. citri* were not affected by this initial lower release rate of fir oil tested. It is also possible that in an absence of choice, *D. citri* may colonize otherwise unacceptable host locations despite the release of otherwise repellent olfactory cues; however, this hypothesis remains to be tested. The physiological need for laying eggs may overwhelm the effect of repellency due to an otherwise unacceptable behavioral cue as also shown with onion fly, *Delia antiqua* (Meigen) [24].

However, in subsequent field-testing, populations of *D. citri* were significantly reduced by fir oil treatment as compared with the control plots. This occurred when the initial fir oil release rate per tree was doubled (8 dispensers/tree). Although it is impossible to directly compare between the field deployment rates of fir oil (4/tree vs. 8/tree) between the two years in this investigation, because they were conducted independently during two consecutive years with varying abiotic conditions, the population densities of *D. citri* were quite similar between years (Figure 3A,B). Therefore, we postulate that the significant reduction in *D. citri* populations observed during the second field experiment may have been the result of doubling the field deployment rate.

However, the cost of the fir oil dispensers used in the field experiments is likely prohibitive (approximately $17/device or $136/tree for the effective treatment) as a stand-alone tool based on the results obtained. Although the higher deployment rate tested in 2014 reduced populations of *D. citri* for two weeks, the cost of insecticides for similar efficacy per hectare is much lower [7]. To become economically viable, a less expensive and more efficient deployment method for fir oil application must be developed. For instance, slow-release nano-dispenser technology that can be sprayed directly onto trees with conventional equipment has been proven to effectively increase the efficiency of insecticide release for management of *D. citri* in citrus with much lower (100 fold) rates of active ingredient needed [25]. Additionally, this tool may be more effective as part of a push-pull or stimulo-deterrent diversion strategy by using the repellent along with trap crop attractants [24]. 

Use of repellents and establishment of effective push-pull management of *D. citri* will be inherently limited by the fact that *D. citri* is an effective vector of *C*Las. Indeed, a single *D. citri* adult is capable of inoculating this disease-causing pathogen into a tree [26]. Repellent, and push-pull systems may only decrease, but not control, HLB infection in commercial citrus production. In the present investigation, we did not measure the level of *C*Las infection before and after the treatment applications because it would have required much larger scale deployment and long-term measurements of infection in the field only possible by establishing a new grove without infection. Therefore these results only provide proof of concept of suppression of vector populations. Much more investigation is required with the entire pathosystem to determine the impact on spread of disease. Furthermore, response of *D. citri* to citrus volatiles is influenced by previous experience with hosts (learning) [27]. It is therefore possible that the effectiveness of olfactory repellents may vary between groves depending on the specific citrus varieties cultivated among different regions. Additionally, it is known that *D. citri* is more attracted toward *C*Las-infected citrus trees than uninfected ones [28]. It is therefore possible that repellents may be less effective on *C*Las-infected trees than on healthy trees. Our future investigations will evaluate long-term efficacy of repellent-based strategies against *D. citri* and their integration with traditional chemical control programs, as well as the effect of such strategies among varying citrus varieties, with different infection statuses. 

The use of repellents against *D. citri* holds promise and it has been documented previously with other active ingredients in the field [29,30]. However, the release technology must be improved. Additionally, integration of botanical oil repellents as part of a broader management strategy for HLB will likely be essential. Currently, the amount of fir oil necessary for efficacy against the vector would be economically prohibitive. However, it is possible that repellents for *D. citri*, may be more useful when integrated with other management tools, such as supplemental insecticides and conservation biological control. The compatibility of botanical oil repellents with biological control agents remains to be investigated for *D. citri* management. 

## 5. Conclusions

Our objective was to determine if the behavior of *D. citri*, a vector of the pathogen causing a fatal disease of citrus, could be manipulated under field conditions with a botanical oil repellent. In the laboratory, *D. citri* were presented with a choice test between control plants and plants treated with the high dose of fir oil. *D. citri* disproportionately settled on control plants, avoiding fir oil-treated plants. However, litsea and citronella oils did not affect behavior of *D. citri* in olfactometer assays. We conducted two field trials to determine if fir oil affected populations of *D. citri* in citrus groves. We found no repellency of *D. citri* from sweet orange resets when treated with high release fir oil devices (10.4 g/day/tree). However, when this deployment dosage was doubled in a subsequent trial, populations of *D. citri* were reduced in treated plots for up to two weeks as compared with untreated controls. Our results suggest that behavior of *D. citri* may not be easily modified under field conditions by olfactory cues from botanical oil treatments alone.

## Figures and Tables

**Table 1:** Response of *Diaphorina citri* to odors of three botanical oils vs. a blank control in a T-olfactometer. Odors of five botanical oils were assayed against clean air in a T-olfactometer to determine the behavioral response of female *D. citri*. The percentage of *D. citri* choosing each odor, as well as the percentage of non-responding *D. citri*, are listed in the table. The corresponding *χ*^2^ values and *p*-values are listed ^a^.

Control Arm	Treatment Arm	*D. citri* Choosing Control Arm (%)	*D. citri* Choosing Treatment Arm (%)	Non Responding *D. citri* (%)	*n*	*χ*^2^ Value	*p*-Value
clean air	Ethylene glycol	41.7	38.3	20.0	60	0.08	0.773
Ethylene glycol	Fir Oil, 1 mg	38.7	45.4	16.0	119	0.64	0.424
Ethylene glycol	Fir Oil, 5 mg	56.3	31.3	12.5	**80**	**5.71**	**0.017 ^†^**
Ethylene glycol	Fir Oil, 15 mg	60.0	16.7	23.3	**60**	**14.70**	**<0.001 ^†^**
Ethylene glycol	Citronella, 1 mg	38.3	50.0	11.7	60	0.93	0.336
Ethylene glycol	Citronella, 5 mg	42.2	36.7	21.1	90	0.35	0.553
Ethylene glycol	Citronella, 15 mg	45.6	36.8	17.5	57	0.53	0.466
Ethylene glycol	Litsea, 1 mg	51.7	43.3	5.0	60	0.44	0.508
Ethylene glycol	Litsea, 5 mg	42.2	50.0	7.8	90	0.59	0.442
Ethylene glycol	Litsea, 15 mg	40.0	28.3	31.7	60	1.20	0.274

^a^ Botanical oil treatments exhibiting significant repellency to *D. citri* are listed in bold and noted with a **^†^**.

**Table 2:** Response of *Diaphorina citri* to fir oil treated citrus vs. untreated citrus in a T-olfactometer. To determine the degree of repellency of fir oil, female *D. citri* were challenged in a T-olfactometer. In the first experiment, odors from fir oil-treated crushed Valencia sour orange leaves were compared to odors from crushed citrus leaves. In the second experiment, odors from a fir oil-treated Valencia sour orange plants were compared to odors from an untreated citrus plants. The percentage of *D. citri* choosing each odor, as well as the percentage of non-responding *D. citri*, are listed in the table. The corresponding *χ*^2^ values and *p*-values are listed ^a^.

Control Arm	Treatment Arm	*D. citri* Choosing Control Arm (%)	*D. citri* Choosing Treatment Arm (%)	Non Responding *D. citri* (%)	*n*	*χ*^2^ Value	*p*-Value
Crushed Citrus	Crushed Citrus + Ethylene glycol	40.0	48.3	11.7	60	0.47	0.492
Crushed Citrus	Fir Oil, 15 mg + Crushed Citrus	43.3	55.0	1.7	60	0.83	0.362
Intact Citrus	Intact Citrus + Ethylene glycol	36.7	43.3	20.0	60	0.33	0.564
Intact Citrus	Fir Oil, 1 mg + Intact Citrus	41.7	30.0	28.3	60	1.14	0.286
Intact Citrus	Fir Oil, 5 mg + Intact Citrus	36.2	36.2	27.6	58	0	1.00
Intact Citrus	Fir Oil, 15 mg + Intact Citrus	53.3	26.7	20.0	60	**5.33**	**0.021 ^†^**

^a^ Botanical oil treatments exhibiting significant repellency at the *p* < 0.05 are listed in bold and noted with a **^†^**.

**Table 3:** Relative peak area (%), and Kovats retention indexes (KRI) of compounds comprising fir oil used in these experiments.

Compounds	KRI	Relative Peak Area (%)
Santene	907	4.11
Tricyclene	921	2.19
α-Pinene	945	19.90
Camphene	964	11.17
2-β-pinene	992	34.29
δ-3-Carene	1018	13.11
para-Cymene	1022	1.58
Unknown	1036	0.29
dl-limonene	1041	6.76
β-Phellandrene	1058	4.82
γ-Terpinene	1062	0.41
Terpinolene	1104	0.47
Bornyl acetate	1297	0.87

## Abbreviations

The following abbreviations are used in this manuscript:

*C*Las	*Candidatus* Liberibacter asiaticus
HLB	huanglongbing
